# Mechanism of the Intermediary Phase Formation in Ti-20 wt. % Al Mixture during Pressureless Reactive Sintering

**DOI:** 10.3390/ma12132171

**Published:** 2019-07-06

**Authors:** Andrea Školáková, Pavel Salvetr, Pavel Novák, Jindřich Leitner, Davy Deduytsche

**Affiliations:** 1 Department of Metals and Corrosion Engineering, University of Chemistry and Technology, Technická 5, 166 28 Prague 6, Czech Republic; 2 Department of Solid State Engineering, University of Chemistry and Technology, Technická 5, 166 28 Prague 6, Czech Republic; 3 Department of Solid State Sciences, Ghent University, Krijgslaan 281 S1 9000 Gent, Belgium

**Keywords:** in situ diffraction, aluminides, reactive sintering, mechanism, powder metallurgy

## Abstract

This work aims to describe the mechanism of intermediary phases formation in TiAl20 (wt. %) alloy composition during reactive sintering. The reaction between titanium and aluminum powders was studied by in situ diffraction and the results were confirmed by annealing at various temperatures. It was found that the Ti_2_Al_5_ phase formed preferentially and its formation was detected at 400 °C. So far, this phase has never been found in this alloy composition during reactive sintering processes. Subsequently, the Ti_2_Al_5_ phase reacted with the titanium, and the formation of the major phase, Ti_3_Al, was accompanied by the minor phase, TiAl. Equations of the proposed reactions are presented in this paper and their thermodynamic and kinetic feasibility are supported by Gibbs energies of reaction and reaction enthalpies.

## 1. Introduction

A Ti-Al system consists of five important phases, including a Ti_3_Al compound with a hexagonal close-packed superlattice (space group P63/mmc), an equiatomic TiAl compound with a tetragonal structure (space group P4/mmm), and aluminum-rich intermetallic compounds, namely TiAl_2_ (space group I41/amd), Ti_2_Al_5_ (space group P4/mmm), and TiAl_3_ (space group I4/mmm), also with a tetragonal structure. All intermetallic compounds are collectively called titanium aluminides. Titanium aluminides belong to the group of innovative materials that gradually replace nickel-based superalloys in highly demanding applications [1]. They possess a great combination of stable mechanical properties at high temperatures (500–900 °C), low density, and good oxidation resistance. For this reason, they are suitable candidates as structural materials for the aerospace and automotive industries. So far, they have been used in turbocharger wheels and turbine blades. The current research is mainly focused on the development of alloys with microstructures containing TiAl and Ti_3_Al phases, which should ensure great creep resistance [1,2,3,4,5]. Despite this advantage, the application range of titanium aluminides is still limited because they suffer from room-temperature brittleness and have poor melt-metallurgic properties [5]. Moreover, the extreme reactivity of molten titanium usually causes contamination of the obtained products [6]. These compounds are produced by a melt-metallurgy process comprising vacuum induction melting (VIM), vacuum arc remelting (VAR), centrifugal casting, conventional melting, and hot isostatic pressing (HIP) [1]. Powder metallurgy techniques are much more suitable because melting is avoided. Thus, molten titanium does not occur, and a high purity of the products can be obtained [1]. One of the currently studied processes of these compounds is Self-propagating High-temperature Synthesis (SHS) which uses solid state diffusion to obtain a mostly local, usually eutectic, composition and a subsequent formation of the liquid phase at temperatures lower than the melting points of the initial materials. This mechanism is, thus, different from reactive sintering using only solid-state diffusion. However, SHS is taken as a specific type of reactive sintering.

The SHS process is a modern method of sintering because time and energy are saved [7,8]. SHS lies in the heating of initial reactants, which are usually powders [9]. Highly exothermic reactions occur during heating [10]. Heat released by these reactions is supported other exothermic reactions, and, thus, the SHS reaction can propagate through the whole heated powder mixture spontaneously [9,11]. For this reason, this reaction is often called as “self-sustaining”. Reactions are usually thermally activated. SHS reaction is divided into two modes, which differ in their heating initiation. A reaction can be initiated by heating of the whole sample, and then it is called Thermal Explosion mode or by heating of only one side of sample and this case of heating is called Ignition mode. The thermal explosion mode usually involves homogeneous heating, while the Ignition mode uses extremely fast heating in most cases [12]. Both modes have their own disadvantages. For the former mode, pre-combustion is necessary and can modify the reaction mechanism. On the other hand, this mode is more accurate in the determination of reaction temperatures. The latter mode is faster, but the results have poor reliability [12].

TiAl and TiAl_3_ aluminides prepared by SHS reaction are the most studied ones [10,13,14,15,16]. Ti_3_Al is not a commonly studied aluminide from the viewpoint of the SHS process, and, hence, its mechanism of phase formation has not yet been described. On the other hand, published research shows contradictory or incomplete results, and the description of the reaction mechanisms of phase formation is not united for TiAl and TiAl_3_ alloy composition. It is known that the reaction between titanium and aluminum powders is initiated when the temperature reaches the melting point of aluminum during heating [17]. The TiAl_3_ phase is concluded to be the first phase during the reaction between liquid aluminum and solid titanium, followed by the formation of the TiAl and Ti_3_Al phases [13,16,17,18,19]. It is believed that the TiAl_3_ phase forms preferentially due to its thermodynamic and kinetic system preferences [20]. The TiAl_2_ phase should form after the formation of the TiAl and Ti_3_Al phases by interdiffusion between the TiAl_3_ phase and titanium [2]. Other works showed that the TiAl phase formed first after a reaction between the melted aluminum and titanium [21]. Further, the TiAl phase is considered to be the starting phase before the formation of the Ti_2_Al_5_ and TiAl_2_ phases [20], followed by the formation of the TiAl_3_ phase. The explanation for the relationship between phases can be found in their low free energies [19]. However, other works showed that a very thin layer of the Ti_3_Al phase formed first on the titanium side surrounded by the TiAl phase and TiAl_3_ formed as the last one [22]. This result shows a completely different phase formation sequence in comparison to the results mentioned before. For this reason, there are holes in the description of reaction mechanisms, and it is necessary to determine the accurate reaction conditions of phase formation.

Ti and Al foils, instead of powders, have also been intensively studied, mainly because of their better mechanical properties. The aim is to synthesize the composite as a sandwich. For this reason, foils are most often produced by electron beam deposition, which affects the phase composition of the interface between aluminum and titanium, and, subsequently, these foils are heated. It was found that diffusion welding formed. Authors usually observe the growth of the intermetallic layer while both reactants are in the solid state and obtained foundational results that they could use determine kinetics. However, they could study only the interface between aluminum and titanium whose chemical composition corresponds to 50:50 in at. % of Ti:Al [23,24,25,26,27]. For this reason, the reaction between solid aluminum and solid titanium is better to simulate the annealing of a compressed powder mixture at temperatures lower than the melting point of aluminum (used in this work). Advantage is possible to mix a different ratio of aluminum to titanium and study Ti_3_Al, TiAl, or TiAl_3_ phases. Moreover, the powder mixture is only compressed, and, thus, the interface between aluminum and titanium is not affected by pre-heating.

In this work, a Ti-20 wt. % Al powder mixture (corresponding to Ti_3_Al compound) was studied. This chemical composition was deliberately chosen because this work is part of project in which the sintering of Ti_3_Al, TiAl, and TiAl_3_ compounds has been studied. The amount of aluminum (20 wt. %) lies in the center of the stability range of the Ti_3_Al phase, according to diagram Ti-Al [28]. This mixture was subjected to the Thermal Explosion mode (TE) SHS, and the mechanism of phase formation was described. In situ diffraction was applied to determine the sequence of phase formation, and their formation was observed at the highest heating rate that the in situ XRD (X-ray diffraction) device allows. To determine whether the phase formed below the melting point of aluminum, powder mixtures were annealed at various temperatures and times. This enabled us to show and confirm the phases’ formation sequence. This work offers the first detailed descriptions of the phase formation of this alloy composition.

## 2. Materials and Methods

All tested samples were prepared from blended powders of titanium (with a purity of 99.5% and a particle size of 44 µm, STREM CHEMICALS, Newburyport, MA, USA) and aluminum (99.62%, 44 µm, STREM CHEMICALS, Newburyport, MA, USA). Powder blends corresponding to the Ti_3_Al compound, i.e., TiAl20 (in wt. %), with a weight of 3 g, were prepared. Subsequently, powder mixture was uniaxially cold pressed at an ambient temperature to cylindrical green bodies of 10 mm in diameter by a pressure of 450 MPa for 5 min using the LabTest 5.260SP1-VM universal loading machine (Labortech, Opava, Czech Republic). Reaction kinetics, especially the initiation of the reactions, strongly depends on the green density of the compacts. We have studied intermetallics for several years and we have experienced that reaction kinetics mainly depend on particle size and compaction pressure. For this reason, we used the finest powders of titanium and aluminum and a compaction pressure of 450 MPa to obtain the powder mixture with the best contacts of particles and the lowest possible porosity. 

Afterwards, the sample was inserted into the induction furnace and heated under a protective Ar atmosphere. Heating was recorded by an optical pyrometer Optris OPTP20-2M (Optris, Portsmouth, NH, USA) to observe the emerging exothermic reaction in the Thermal Explosion (TE-SHS) mode. The heating rate was determined, according to the slope of the obtained curve, as 109 °C·min^−1^. In order to describe the phases’ formation sequence during the SHS process, in situ XRD analysis (Department of Solid State Sciences, Ghent University, Ghent, Belgium) of the compressed powder mixture was performed under an He atmosphere during heating to 900 °C, with a heating rate of 60 °C·min^−1^. XRD source and linear Vantec detector were fixed at the positions of 21° (source) and 42° (detector).

Further, the formation of phases was observed at temperatures lower than the melting point of aluminium −400, 450, 500, and 600 °C. Samples were evacuated in silica ampoules and exposed at these temperatures for 8, 24, and 48 h.

The obtained samples were ground by sandpaper P80–P4000 with SiC abrasive particles (Hermes Schleifmittel GmbH, Hamburg, Germany), polished by suspension of colloidal silica Eposil F (ATM GmbH, Mammelzen, Germany) with hydrogen peroxide (volume 1:6), and etched by Kroll´s reagent (5 mL HNO_3_, 10 mL HF, 85 mL H_2_O). The microstructure was examined by a scanning electron microscope TESCAN VEGA 3 LMU (Tescan, Brno, Czech Republic) equipped with an OXFORD Instruments X-max EDS SDD 20 mm^2^ detector (Oxford Instruments, High Wycombe, UK) for identification of the chemical composition of individual phases (SEM-EDS). Phase composition was determined by X-ray diffraction using the PANalytical X´Pert Pro diffractometer with CuKα radiation (PANalytical, Almeo, The Netherlands).

## 3. Results and Discussion

**Figure 1:**
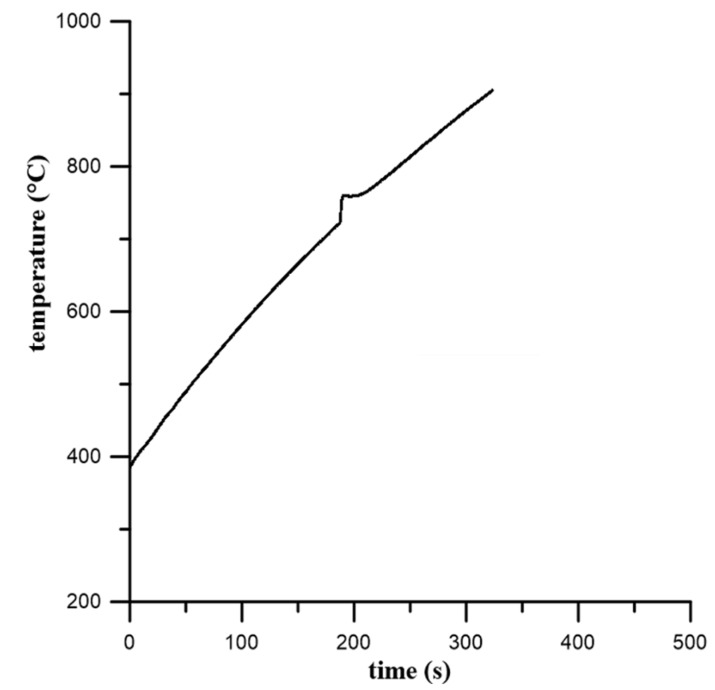
Heating curve obtained by the optical pyrometer at a heating rate of 109 °C·min^−1^.

**Figure 2:**
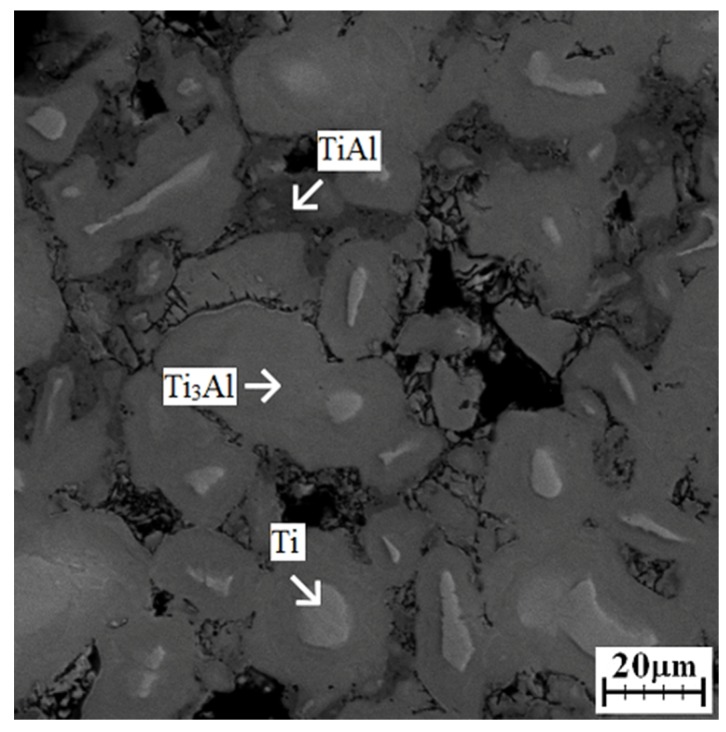
SEM image of microstructure obtained after induction at heating rate of 109 °C·min^−1^.

**Figure 3:**
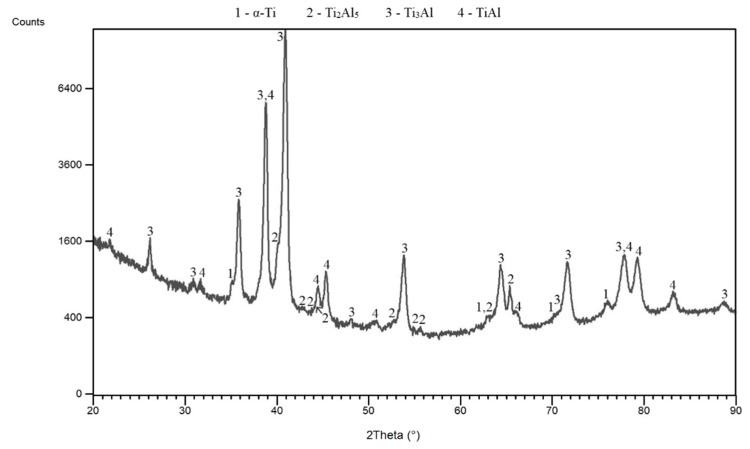
XRD patterns obtained after induction at a heating rate of 109 °C·min^−1^.

Figure 1 shows the heating curve obtained during the reactive sintering of the Ti-20 wt. % Al powder mixture in an induction furnace. The exothermic peak is associated with the SHS reaction and formation of phases. The reaction started above the temperature of the melting point of aluminum because T_onset_ was 716 °C, indicating that the presence of a liquid phase triggered the reaction. This means that the reaction was not initiated at the melting point of aluminum at 660 °C, as is typical for aluminides [16]. The liquid phase was present, accelerated the sintering at higher temperatures, and was gradually consumed during the process [2]. The maximum SHS reaction, called the combustion temperature, was 760 °C. The microstructure was composed of unreacted titanium, which was surrounded by large areas of the Ti_3_Al phase (Figure 2). TiAl was found between the layers of the Ti_3_Al phase. These phases were also detected by XRD analysis (Figure 3), and, moreover, the Ti_2_Al_5_ phase was found. This phase is very fine and is probably dispersed in the matrix uniformly. According to the area fraction, it can be assumed that the Ti_3_Al phase formed during an exothermic SHS reaction, which was observed by an optical pyrometer (Figure 1). This phase also formed as a major phase in Ti:Al = 1:1 system [16], and it was stated that its formation is associated with diffusion. On the other hand, the Ti_2_Al_5_ phase was not detected in this aluminum enriched system [16]. Results from the EDS analysis are shown in Table 1.

**Figure 4:**
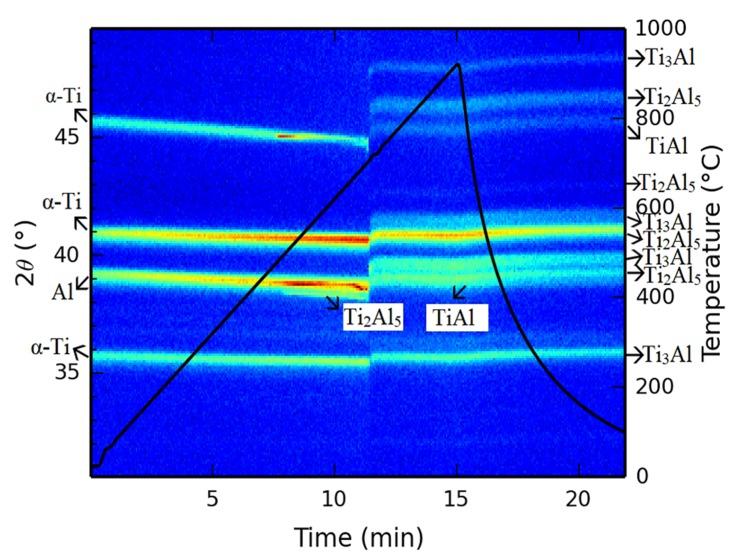
In situ diffraction obtained during heating (heating rate of 60 °C·min^−1^).

The in situ XRD analysis shows the continuous formation of phases from the powder mixture during heating (Figure 4). This observation of phase composition during heating helps to describe the mechanisms of phase formation. It can be seen that titanium and aluminum were present up to approximately 500 °C. At this temperature, the Ti_2_Al_5_ phase started to form. Titanium and aluminum diffraction lines disappeared at approximately 700 °C. This disappearance is associated with the formation of other phases—Ti_3_Al and TiAl. These phases were accompanied by the Ti_2_Al_5_ phase, which had formed earlier. It was impossible to determine which of the phases (Ti_3_Al or TiAl) formed preferentially. All their diffraction lines appeared at the same temperature, i.e., approximately 700 °C. The intensity of the lines of the Ti_2_Al_5_ phase was distinctive from 500 °C to 700 °C. This result implies that the SHS reaction between the titanium and aluminum powders is initiated by the formation of an intermediary Ti_2_Al_5_ phase. Many works [2,16,17,18,19,20] claimed that the TiAl_3_ phase formed before the formation of the TiAl, Ti_3_Al, or TiAl_2_ phases already at 580 °C. Their explanation as to why the TiAl_3_ phase arose preferentially lies in its minimum free energy, suggesting that its formation is thermodynamically and kinetically favored over the formation of other aluminides. Only the presence of liquid aluminum is sufficient for the formation of the TiAl_3_ phase [20]. On the other hand, in situ XRD analysis, and our other results, showed that the Ti_2_Al_5_ phase is formed preferentially in a TiAl20 powder blend. The reaction temperature is an important parameter that can support or induce synthesis of aluminides, as described in work [29]. Based on the presented results, the other part of this study focused on individual temperatures to clarify aluminide formation.

**Figure 5:**
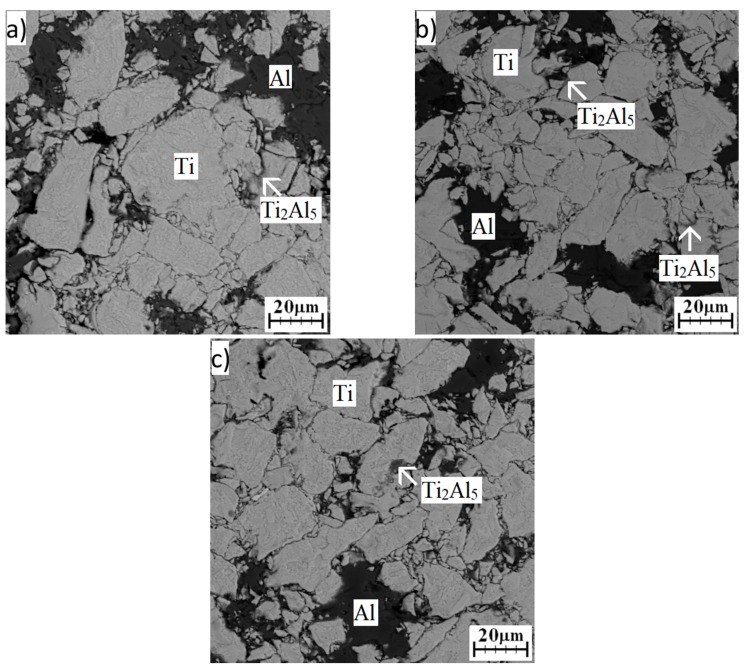
SEM images of a microstructure of a TiAl20 powder mixture annealed at 400 °C: (**a**) 8 h; (**b**) 24 h; (**c**) 48 h.

**Figure 6:**
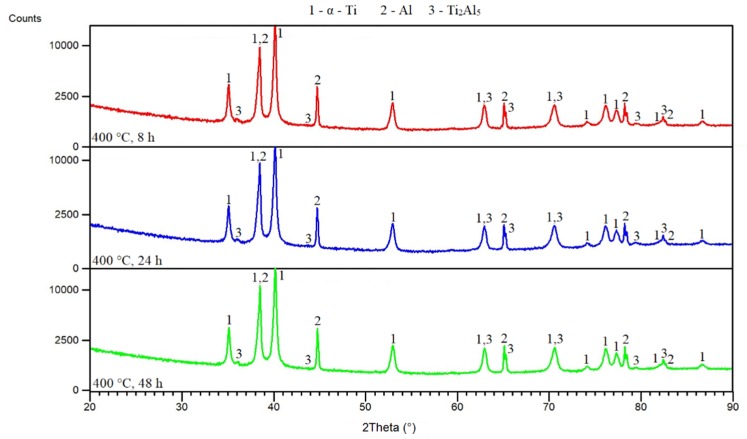
XRD patterns of TiAl20 powder mixture annealed at 400 °C.

From the obtained results, it can be seen that the Ti_2_Al_5_ phase formed below the melting point of aluminum. For this reason, mixtures of powders were annealed at various temperatures, and times, microstructures, and phase compositions were observed. Figure 5a–c shows microstructures obtained after annealing at 400 °C. Unreacted particles of aluminum and titanium were found. However, XRD analysis (Figure 6) revealed that Ti_2_Al_5_ formed at this temperature, and this phase was observed at the interface of titanium and aluminum. In situ XRD analysis detected its formation at 500 °C, but analysis was performed at a heating rate of 60 °C·min^−1^ (the maximum heating rate that can be set), and it is known that with a decreasing heating rate, the initiation temperatures also decrease [30]. Moreover, the heating during the in situ XRD experiment was rather quick and continuous. Therefore, the amount in the Ti_2_Al_5_ phase, which is expected to form by a diffusion mechanism, is very low during continuous heating, and, therefore, it may not be detected. During annealing, the detectable amount of the Ti_2_Al_5_ phase was formed after 8 h at 400 °C (Figure 6). The EDS results are listed in Table 2. It can be seen that chemical composition does not correspond to Ti_2_Al_5_ but the analyzed area is small and, thus, strongly affected by titanium.

**Figure 7:**
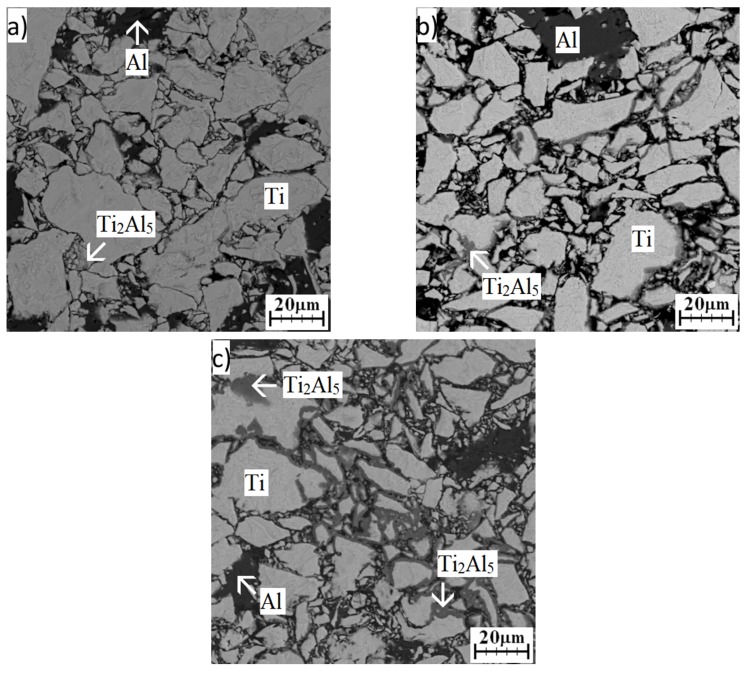
SEM images of the microstructure of the TiAl20 powder mixture annealed at 450 °C: (**a**) 8 h; (**b**) 24 h; (**c**) 48 h.

**Figure 8:**
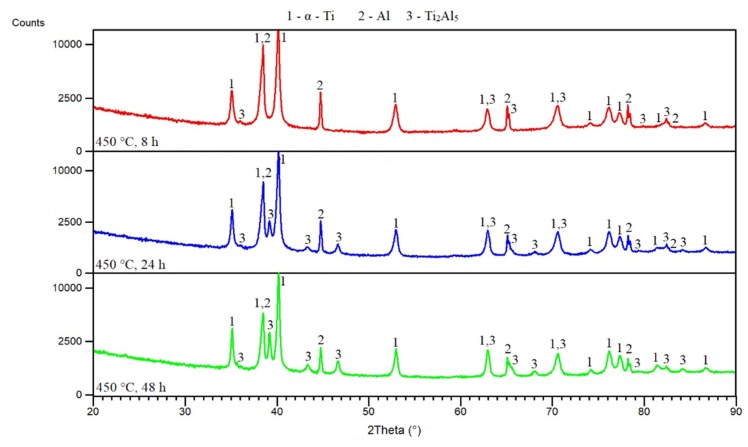
XRD patterns of TiAl20 powder mixture annealed at 450 °C.

Annealing at a higher temperature (450 °C) for 8 h allowed the Ti_2_Al_5_ phase to be observed in the microstructure more markedly (Figure 7a). The microstructure was, again, composed of unreacted particles. One day annealing affected the microstructure significantly. The Ti_2_Al_5_ phase was detected by EDS analysis (see Table 3) on the interface between the titanium and aluminum powders (Figure 7b). With prolongation of annealing time, the areas of occurrence of the Ti_2_Al_5_ phase were larger (Figure 7c). XRD analysis confirmed the presence of hexagonal titanium, aluminum, and the Ti_2_Al_5_ phase (Figure 8). 

**Figure 9:**
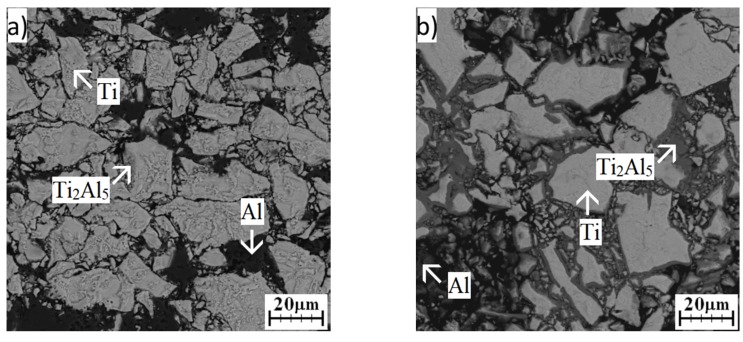

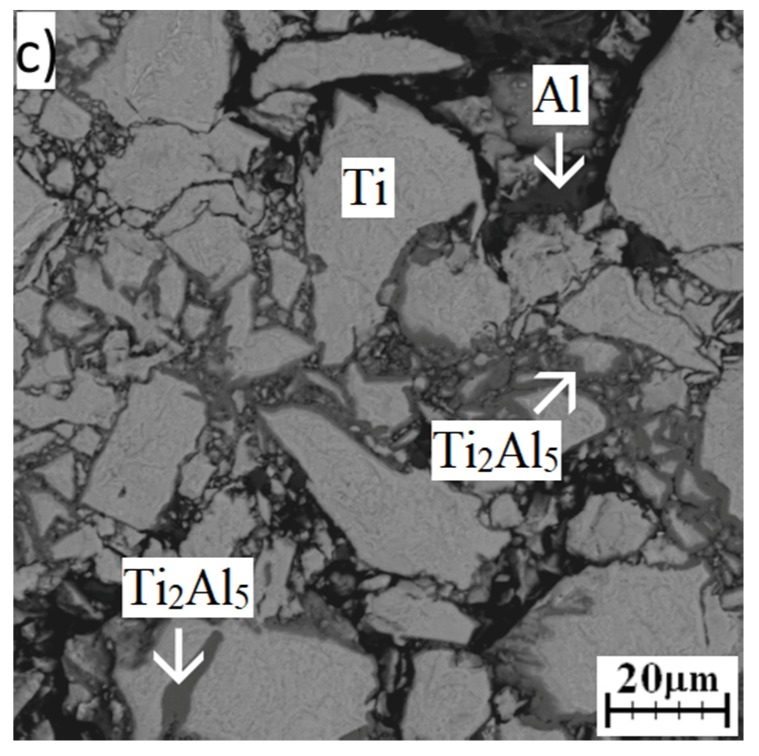
SEM images of the microstructure of TiAl20 powder mixture annealed at 500 °C: (**a**) 8 h; (**b**) 24 h; (**c**) 48 h.

**Figure 10:**
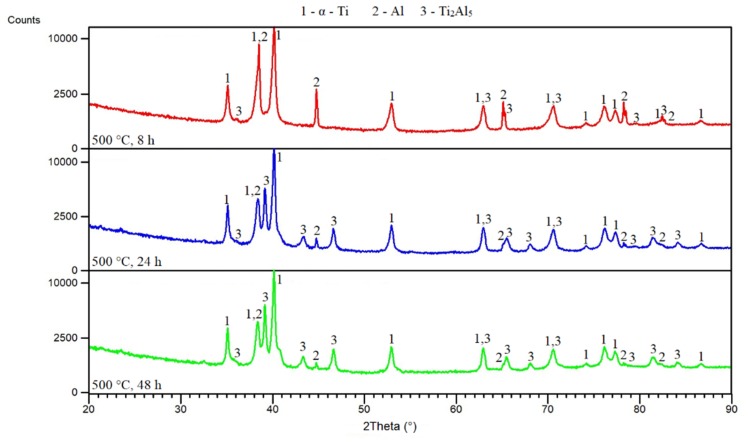
XRD patterns of TiAl20 powder mixture annealed at 500 °C.

The area of the Ti_2_Al_5_ phase was found during EDS analysis of the microstructure after annealing at 500 °C for 8 h (Figure 9a), but prolongation of times clearly revealed its location. The Ti_2_Al_5_ phase formed on the interfaces between aluminum and titanium particles, as well as at lower temperatures (Figure 9b,c). EDS analysis also confirmed its presence (Table 4). The phase composition was confirmed by XRD analysis (Figure 10).

**Figure 11:**
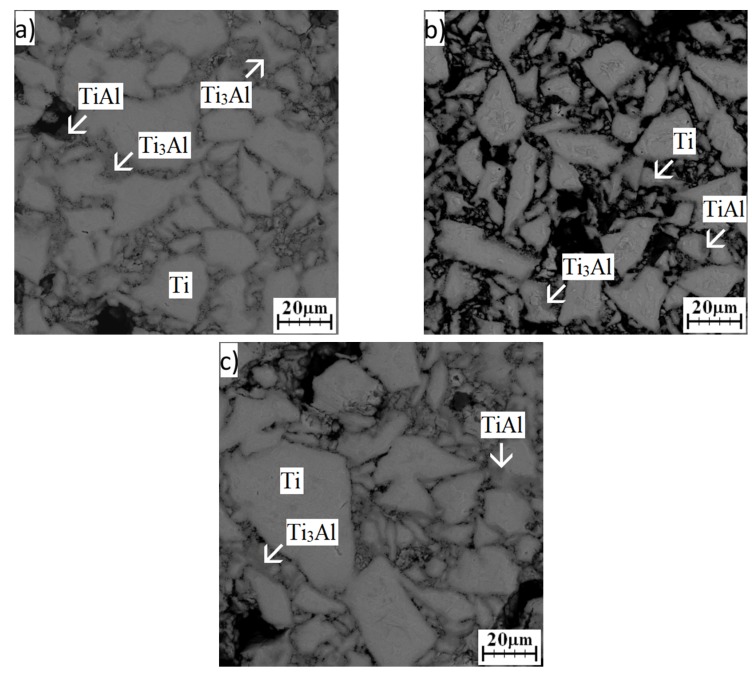
SEM images of the microstructure of TiAl20 powder mixture annealed at 600 °C: (**a**) 8 h; (**b**) 24 h; (**c**) 48 h.

**Figure 12:**
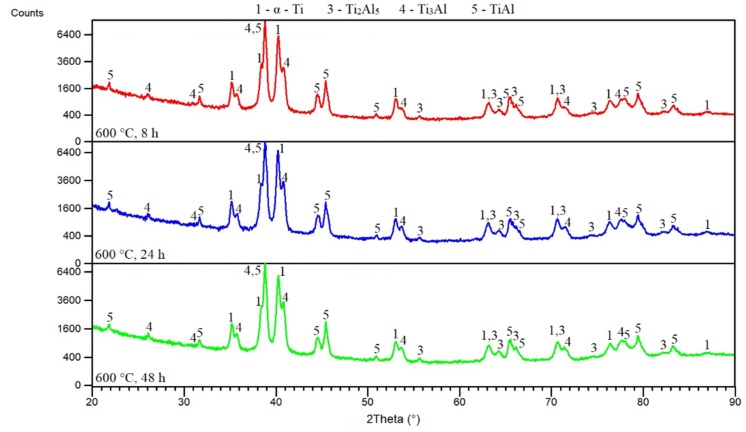
XRD patterns of TiAl20 powder mixture annealed at 600 °C.

A temperature of 600 °C changed the microstructure significantly. Unreacted aluminum was not detected after all annealing times (Figure 11a–c). The Ti_3_Al and TiAl phases were determined by a combination of EDS (Table 5) and XRD analysis (Figure 11a–c and Figure 12). Ti_3_Al replaced the Ti_2_Al_5_ phase and formed an interface between the original titanium particles. The TiAl phase was found only in some interfaces, and these interfaces were always darker (Figure 11a–c). In situ diffraction (Figure 4) and the results from microstructure observation showed that the Ti_3_Al and TiAl phases formed simultaneously, but the Ti_3_Al phase is the major phase. XRD analysis detected the Ti_2_Al_5_ phase, which was still present and did not disappear after formation of phases enriched by titanium (Figure 12). Ti_2_Al_5_ occurring in the microstructure was not detected by EDS analysis, likely due to its small dimensions and low volume fraction. This means that the Ti_3_Al phase really formed as the main phase during the SHS reaction. Its formation was accompanied by the formation of the minor phase, TiAl. The Ti_2_Al_5_ phase formed preferentially as a metastable reaction intermediate. The Ti_2_Al_5_ and TiAl phases both have a tetragonal structure [2], and this could be one of the reasons why the TiAl phase could be stabilized as a minor phase.

The probable reaction scheme, with the Gibbs energies of reaction and reaction enthalpies, is as follows (Equations (1)–(3)):

2 Ti + 5 Al → Ti_2_Al_5_,
∆H (400 °C) = −271.06 kJ,
∆G (400 °C) = −226.29 kJ (1)

Ti_2_Al_5_ + 13 Ti → 5 Ti_3_Al,
∆H (600 °C) = −293.81 kJ,
∆G (600 °C) = −242.84 kJ (2)

Ti_2_Al_5_ + 3 Ti → 5 TiAl,
∆H (600 °C) = −72.38 kJ,
∆G (600 °C) = 15.75 kJ (3)

Our calculations are derived from the thermodynamic data published in [31,32].

## 4. Conclusions

According to the obtained results, the mechanism of phase formation in Ti-Al systems can be described. The SHS reaction started at temperatures higher than the melting point of aluminum. It was found that Ti_2_Al_5_ phase formed preferentially, even though a titanium-rich powder mixture was investigated. The Ti_2_Al_5_ phase formed at 400 °C by a diffusion-controlled reaction. This phase subsequently reacted with the titanium, and the Ti_3_Al and TiAl phases formed simultaneously. These two phases could form only above 600 °C. The Ti_3_Al phase was a major phase. The results were compared between the data obtained by in situ diffraction and the annealed samples, and the described mechanism was confirmed.

## Figures and Tables

**Table 1:** SEM + EDS point analysis results of the reactively sintered TiAl20.

Phases	Element Concentration (at. %)	
	Ti	Al
TiAl	50 ± 2	50 ± 2
Ti_3_Al	72 ± 3	28 ± 3

**Table 2:** SEM + EDS point analysis results of TiAl20 annealed at 400 °C.

Phase	Element Concentration (at. %)	
	Ti	Al
Ti_2_Al_5_, 48 h	42 ± 3	58 ± 3

**Table 3:** SEM + EDS point analysis results of TiAl20 annealed at 450 °C.

Phase	Element Concentration (at. %)	
	Ti	Al
Ti_2_Al_5_, 8 h	38 ± 10	62 ± 10
Ti_2_Al_5_, 24 h	41 ± 4	59 ± 4
Ti_2_Al_5_, 48 h	30 ± 3	70 ± 3

**Table 4:** SEM + EDS point analysis results of TiAl20 annealed at 500 °C.

Phase	Element Concentration (at. %)	
	Ti	Al
Ti_2_Al_5_, 8 h	30 ± 10	70 ± 10
Ti_2_Al_5_, 24 h	32 ± 7	68 ± 7
Ti_2_Al_5_, 48 h	35 ± 3	65 ± 3

**Table 5:** SEM + EDS point analysis results of TiAl20 annealed at 600 °C.

Phases	Element Concentration (at. %)	
	Ti	Al
TiAl, 8 h	51 ± 6	49 ± 6
Ti_3_Al, 8 h	64 ± 4	36 ± 4
TiAl, 24 h	50 ± 6	50 ± 6
Ti_3_Al, 24 h	65 ± 3	35 ± 3
TiAl, 48 h	48 ± 5	52 ± 5
Ti_3_Al, 48 h	71 ± 7	29 ± 7
